# Role of p53 in pseudorabies virus replication, pathogenicity, and host immune responses

**DOI:** 10.1186/s13567-019-0627-1

**Published:** 2019-02-04

**Authors:** Xun Li, Wei Zhang, Yunjia Liu, Jiaxun Xie, Chuanhuo Hu, Xiaoye Wang

**Affiliations:** 1 College of Animal Science and Technology, Guangxi University, Nanning, 530004 Guangxi People’s Republic of China; 2 Department of Biochemistry and Molecular Biology, School of Basic Medical Sciences, Nanjing Medical University, Nangjing, 211166 People’s Republic of China

## Abstract

As a key cellular transcription factor that plays a central role in cellular responses to a broad range of stress factors, p53 has generally been considered as a host cell restriction factor for various viral infections. However, the defined roles of p53 in pseudorabies virus (PRV) replication, pathogenesis, and host responses remain unclear. In the present study, we initially constructed a p53 overexpressing a porcine kidney epithelial cell line (PK-15) to detect the effect of p53 on PRV replication in vitro. The results show that viral glycoprotein B (gB) gene copies and the titers of virus were significantly higher in p53 overexpressing PK-15 cells than in PK-15 and p53 inhibitor treated p53 overexpressing PK-15 cells. A similar result was also found in the p53 inhibitor PFT-α-treated PK-15 cells. We then examined the effects of p53 on PRV infection in vivo by using p53-knockout (p53^−/−^) mice. The results show that p53 knockout not only led to significantly reduced rates of mortality but also to reduced viral replication and development of viral encephalitis in the brains of mice following intracranial inoculation. Furthermore, we examined the effect of p53 knockout on the expression of the reported host cell regulators of PRV replication in the brains of mice by using RNA sequencing. The results show that p53 knockout downregulated the interferon (IFN) regulator genes, chemokine genes, and antiviral genes after PRV infection. This finding suggests that p53 positively regulates viral replication and pathogenesis both in vitro and in vivo. These findings offer novel targets of intrinsic host cell immunity for PRV infection.

## Introduction

Pseudorabies virus (PRV) belongs to the genus *Varicellovirus* in the subfamily Alphaherpesvirinae and it is the pathogen that causes porcine Aujeszky’s disease (AD) [1]. PRV causes nervous and respiratory system disorders in newborn piglets and reproductive failure in sows [2]. The virus has a broad host range and can infect most mammals; however, pigs are the natural reservoir and the only animal that can survive PRV infection [1]. The clinical manifestations of other animals infected by PRV are fatal and acute, and accompanied by extreme itching.

The tumor suppressor protein p53 is a major host cellular response protein to a broad range of stress factors such as viral infection through its modulation of cellular pathways, including innate immune control, host cell cycling, proliferation, DNA repair, and apoptosis [3–5]. Viral infection is a type of cellular stress that activates p53 response that triggers apoptosis of the infected cells, leading to the suppression of viral replication [6–8]. Thus, p53 is considered as a host restriction factor in a range of viral infections. However, p53 appears to have both positive and negative effects on various viral infections. The replication of various viruses is enhanced by the knockout or knockdown of p53 and inhibited by the overexpression of p53. Examples of such viruses include hepatitis C virus (HCV), influenza A virus (IAV), Japanese encephalitis virus (JEV), and vesicular stomatitis virus (VSV) [6, 8, 9]. Furthermore, many viruses have acquired a variety of distinct mechanisms to counteract the negative effects of p53 in infected cells [6]. Conversely, p53 is required for efficient viral replication of other viruses. p53 knockdown impairs the replication of herpes simplex virus 1 (HSV-1) and the associated viral pathogenesis of the central nervous system (CNS) of mice [10, 11]. It was also reported that the replications of human cytomegalovirus (HCMV) and porcine circovirus type 2 were impaired by p53 knockdown [12, 13].

Collectively, the studies described above indicate that p53 is a critical host restriction factor for a variety of viruses. However, there are no studies that have examined the effects of p53 on PRV infection. The biological significance of p53 in PRV replication, pathogenicity, and host immune responses remains to be elucidated. In the present study, we investigated the role(s) of p53 in PRV replication by using p53 overexpressing PK-15 cells and the p53 inhibitor PFT-α-treated PK-15 cells in vitro. Furthermore, we investigated the effects of p53 on the replication and pathogenesis of PRV in vivo by using p53 knockout mice. The primary aim of this study was to elucidate the underlying mechanisms responsible for the role of p53 involvement in the replication and pathogenesis of PRV and to offer novel targets of intrinsic host cell immunity for PRV infection.

## Materials and methods

### Cells, mice, and virus

The PK-15 cell line was purchased from the American Type Culture Collection (ATCC, Rockville, MD, USA). The cells were grown in Dulbecco’s Modified Eagle’s medium (DMEM, Wisent) supplemented with 10% fetal bovine serum (FBS, Invitrogen, Carlsbad, CA, USA) and 80 μg of gentamycin/mL at 37 °C in a humidified atmosphere of 5% CO_2_.

A p53 overexpressing PK-15 cell line (PK-15 pCDH-p53) was constructed and maintained in our laboratory. The p53 gene (GenBank No.AF098607.1) was synthesized and cloned into pCDH-CMV-MCS-EF1-CopGFP-T2A-Puro (pCDH-MCS-GFP-Puro) to generate the recombinant lentiviral vector pCDH-CMV-p53-EF1-CopGFP-T2A-Puro (pCDH-p53-GFP-Puro). The insertion fragment was identified by polymerase chain reaction (PCR), restriction endonuclease analysis, and DNA sequencing. The plasmid lentiviral vector system was transfected into PK-15 cells with Lipofectin 2000 reagent for packaging into mature lentivirus. Polybrena was used to screen stably expressing p53 PK-15 cells. An empty lentiviral vector was also transfected into PK-15 cells as the negative control (PK-15 pCDH). After 48 h of transfection, the cells were collected and stored at −80 °C until the expression level of p53 was measured by real-time PCR and Western blot.

p53 knockout (p53^−/−^) mice and wild-type mice littermates were obtained by interbreeding heterozygous mice (stock number B-EM-020), provided by the Beijing Biocytogen Co., Ltd.

The PRV strain Bartha K61 was isolated and purified from vaccine (Jiangsu Nannong Gaoke Animal Pharmaceutical Co., Ltd.) and maintained in our laboratory. PK-15 was used for the propagation of PRV.

### Cell studies

PK-15 p53^+/+^ cells were infected with 0.1 multiplicity of infection (MOI) PRV. At 8, 12, 24, and 48 h post-infection, the cells were harvested to detect the viral titer, and glycoprotein B (gB) mRNA expression of PRV utilized real-time PCR and 50% tissue culture infective dose (TCID_50_), respectively.

PK-15 cells were treated with PFT-α (p53 inhibitor) and then infected with 0.1 MOI of PRV. The cells were also harvested to detect the gB mRNA expression of PRV by real-time PCR at 8, 12, 24, and 48 h post-infection.

### Animal studies

All the animal experiments and the protocols used in this study were approved by the Research Ethics Committee of the College of Animal Science, Guangxi University, Guangxi, China. The animals were maintained under constant conditions of light (12 h of light) and temperature (22 °C) and housed in groups of four mice per cage until the beginning of the tests. The mice, had free access to pelleted food and tap water. For intracranial infection, 4- to 8-week-old male p53^−/−^ mice (*n* = 40) and wild-type mice (*n* = 40) were injected intracranially with 10^5^ TCID_50_ of PRV (Bartha). Fifteen mice among the total of either p53^−/−^ or wild-type mice were monitored daily, and the mortality was recorded from 1 to 14 days post-infection. Twenty-five mice among the total p53^−/−^ or wild-type mice were killed at 6 days after infection by decapitation under a mild dose of anesthetic ether within 30 min. The brains were excised and cleaned. Twelve brains of p53^−/−^ or wild-type mice were stored at −80 °C to detect the host cellular gene expression by RNA sequencing. Six brains of p53^−/−^ or wild-type mice were stored at −80 °C to detect the viral titer as well as the expression of the gB gene and inflammatory factors by using TCID_50_ and real-time PCR, respectively. Blood serum was also collected and kept at 4 °C to detect the inflammatory factor through specific antibodies by enzyme-linked immunosorbent assay (ELISA). The other 7 brains of p53^−/−^ or wild-type mice were fixed in neutral-buffered formalin for histological analysis.

### Histopathology

For histological analysis, the brains were dehydrated in an ethanol series and embedded in paraffin wax, followed by histologic sectioning (5 μm) and routine hematoxylin–eosin staining. To describe the histopathology in the brain in the p53^−/−^ and p53^+/+^ mice, the pathological changes were determined through observation of the morphologic characteristics. For this purpose, 5 slides were selected from 1 sample, and 2 sections from each slide were examined microscopically (magnification: ×400).

### RNA extraction and real-time reverse transcription PCR (RT-PCR)

Total RNA from the brains or cells was extracted using the TRIzol extraction method (TRIzol reagent, TaKaRa Japan). The procedures for RNA isolation and purification, as well as the on-column deoxyribonuclease treatment (Qiagen), were performed according to the manufacturer’s instructions. For each sample, equal amounts of all the RNA samples were reverse transcribed simultaneously using an oligo (deoxythymidine) 15 primer and M-MLV reverse transcriptase (TaKaRa Japan) according to the manufacturer’s instructions. All RT reactions were performed at 42 °C and included a negative control, which contained nuclease-free water instead of RNA. The SYBR Green Quantitative Real-Time PCR Master Mix (Roche, Mannheim, Germany) was used to quantify or relatively quantify the abundance of the target mRNA according to the manufacturer’s instructions, and the accumulated fluorescence was detected using a real-time PCR detection system (Prism 7300, Applied Biosystems Inc., Foster City, USA). The primer sequences are shown in Table 1. β-Actin served as the endogenous control. The real-time PCR amplification conditions were as follows: initial denaturation at 95 °C for 10 min, followed by 40 cycles of 95 °C for 20 s, annealing for 30 s at 59–61 °C, and then extension at 72 °C for 30 s. To quantify the viral load, the PRV viral gB mRNA level was determined by absolute quantification real-time PCR. The gB gene was constructed from the pMD-19T vector (Promega, Madison, WI, USA) and termed pMD-gB. A standard graph of the C_T_ values was constructed from a tenfold serial dilution of the pMD-gB. The C_T_ values from the test samples were plotted on the standard curve, and the copy number was calculated automatically by Sequence Detector version 1.6 (PE Applied Biosystems), a software package for data analysis. Each sample was tested in duplicate, and the mean of the two values was considered as the copy number of the sample. The expression levels of the other target genes were measured by relative quantitative real-time PCR. The data for each sample were calculated using the 2^−ΔΔCT^ method as described previously.

**Table 1:** **Primers and annealing temperature for real-time PCR.**

Genes	Primer sequence (5′-3′)	Annealing (°C)
gB	(F)CGGCATCGCCAACTTCTTC (R)GTCCTCCTTGAGCGTCTTCGT	61
IL-6	(F)AGTCCGGAGAGGAGACTTCA (R)ATTTCCACGATTTCCCAGAG	60
TNF-α	(F)GGGACAGTGACCTGGACTGT (R)GCTCCAGTGAATTCGGAAAG	61
Oasl2	(F)ACAATTTCCAAAACGAGGTC (R)TTCCCATCCCTTTCTTCTTC	59
Ifi44	(F)GACAGATACCAGTTCGATTC (R)TTTTCTTGATCTTTGCCACC	60
Usp18	(F)CAAGGAACAGTCTGAAATACAC (R)CACAGTAATGACCAAAGTCAG	60
Ifit1	(F)AGAACAGCTACCACCTTTAC (R)TTCTTGATGTCAAGGAACTG	61
Ly6a	(F)GAGAGGAAGTTTTATCTGTGC (R)TCTCAAATGGGACTCCATAG	60
Gbp10	(F)CTAACCGGAAGTGTTTTGTC (R)CAGAATCCCTAGTTTATTCCC	59
Gbp4	(F)AGCTAACGAAGGAACAAAAG (R)GATGTTATGTCCCAGTTGATG	60
Gbp3	(F)CTGTTCGAGATTTTGCTCTG (R)TGGACTTTGAGATTGTCTCC	60
Gbp7	(F)GAGTGAAGGCAAATCATGTC (R)CTGTTTCTGTCTTAGTAGCTC	60
Stat1	(F)TTTGACAGTATGATGAGCAC (R)AGCAAATGTGATGCTCTTTC	61
Ddx58	(F)GAGAGTCACGGGACCCACT (R)CGGTCTTAGCATCTCCAACG	61
Ifih1	(F)TGATGCACTATTCAAGAACTAACA (R)TCTGTGAGACGAGTTAGCCAAG	59
Ifitm3	(F)TCATCATTGTTCTTAACGCTCA (R)CGGAAGTCGGAATCCTCTAT	60
Mx1	(F)GAAGGCAAGGTCTTGGATG (R)GCTGACCTCTGCACTTGACT	60
Ifi44	(F)TTCAACTCAGTGGAAGTCTGCT (R)GGAGTGTTTCCCCGCTTTTTC	61
Ccl2	(F)TCTGTGCTGACCCCAAGAAGG (R)TGGTTGTGGAAAAGGTAGTGGAT	59
Cxcl10	(F)TCCCTCTCGCAAGGAC (R)TTGGCTAAACGCTTTCAT	59
β-actin	(F)AGGTGACAGCATTGCTTCTG (R)GCTGCCTCAACACCTCAAC	60

### RNA sequencing

For RNA sequencing analysis, equal amounts of the total RNA from either the p53^−/−^ or wild-type mice (*n* = 12) were pooled into one sample. The procedure used for RNA sequencing has been described previously. Briefly, sequencing libraries were constructed with the SureSelect Strand-Specific RNA library (Agilent); 100-bp paired-end sequencing was performed using an Illumina HiSeq 2500 sequencer according to the manufacturer’s instructions. The raw sequence reads were mapped to the mouse genome by using the TopHat program. The normalized transcription profiles were estimated on the basis of the mapping results using the Cufflinks program.

The number of reads per kilobase of exon per million mapped reads (RPKM) was converted from the row read counts of each transcript using the program Cuffdiff.

### Elisa

Levels of the cytokines interleukin (IL)-6 and tumor necrosis factor (TNF)-α were measured by ELISA using the mouse IL-6 and TNF-α kits (Biosource). Blood samples were collected, and serum was separated. The serum was then added to wells coated with monoclonal antibodies against IL-6 and TNF-α. After 3 washes with washing buffer (0.05% Tween-20 in phosphate-buffered saline, PBS), peroxidase-conjugated avidin, biotinylated antibodies against IL-6 and TNF-α, and chromogenic substrates were added to each well. The absorbance was read at 450 nm in an ELISA plate reader.

### Virus infection and titration

PRV was propagated in PK-15 cells in DMEM supplemented with 10% FBS until harvested. Then, viral stock titers were measured using the plaque-forming unit (PFU) assay, and TCID_50_ was calculated using the Reed-Muench method.

### Statistical analysis

All the data are shown as mean ± S.E.M. The differences were considered to be significant when *P* < 0.05. Statistical analysis was performed using one-way analysis of variance (ANOVA) with SPSS 17.0.

## Results

### p53 facilitates PRV viral replication in vitro

**Figure 1:**
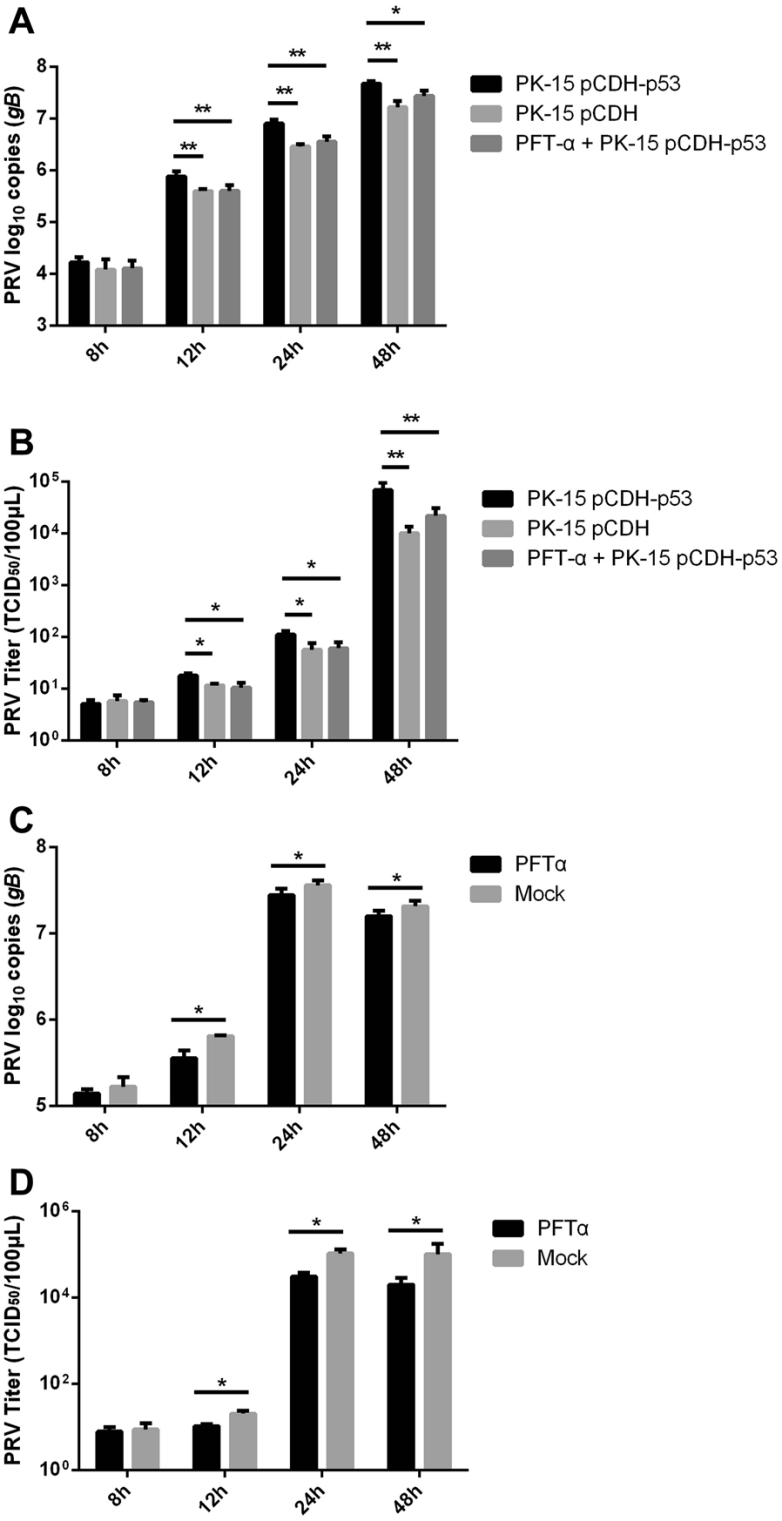
**Effect of p53 on the PRV replication in vitro**. **A** The viral gB gene copies were detected in PFT-α (p53 inhibitor) treated PK-15 pCDH-p53, PK-15 pCDH-p53 and PK-15 pCDH cells after 0.1 MO PRV infection in 4, 8, 12, and 24 h by quantitative real-time PCR. **B** Titers of PRV were determined in PFT-α treated PK-15 pCDH-p53, PK-15 pCDH-p53 and PK-15 pCDH cells after 0.1 MO PRV infection in 4, 8, 12, and 24 h by TCID_50_. **C** The viral gB gene copies were detected in PFT-α treated PK-15 and mock PK-15 cells after 0.1 MO PRV infection in 4, 8, 12, and 24 h by quantitative real-time PCR. **D** Titers of PRV were determined in PFT-α treated PK-15 and mock PK-15 cells after 0.1 MO PRV infection in 4, 8, 12, and 24 h by TCID_50_. All the results were confirmed by three independent experiments. Error bars represent the standard deviations of triplicate experiments. *P < 0.05; **P < 0.01.

gB is the most conserved envelope glycoprotein across the herpesvirus family. Because of its indispensable role in fusion during virus entry and cell-to-cell viral spread, it is required for viral replication. To investigate the effect of p53 on PRV replication in vitro, we estimated the viral load and virus yield by quantification real-time PCR and TCID_50_ assays in PFT-α (p53 inhibitor) treated PK-15 pCDH-p53 and PK-15 pCDH-p53 cells compared with PK-15 pCDH. As shown in Figure 1A, the viral gB gene copies gradually increased from 4 h to 24 h in PFT-α treated PK-15 pCDH-p53, PK-15 pCDH-p53 and PK-15 pCDH cells, but the viral load in PK-15 pCDH-p53 cells exceeded that in PK-15 pCDH and PFT-α treated PK-15 pCDH-p53 cells in 8 h, 12 h, and 24 h. The viral gB gene copies show no significant difference between PFT-α treated PK-15 pCDH-p53 and PK-15 pCDH cells. The results of viral titers were consistent with the viral load test (Figure 1B). To further confirm the promotive effect of p53 on PRV viral replication, PK-15 cells treated with PFT-α were also used to estimate the virus yield after PRV infection compared with mock PK-15 cells. The results show that PFT-α-treated PK-15 cells exhibited significantly lower viral gB gene copies (Figure 1C) and viral titers (Figure 1D) than mock PK-15 cells. These results indicate that p53 facilitates PRV viral replication by increasing gB expression and PRV progeny yields in vitro.

### p53 promoted the PRV replication in mice brain in vivo

**Figure 2:**
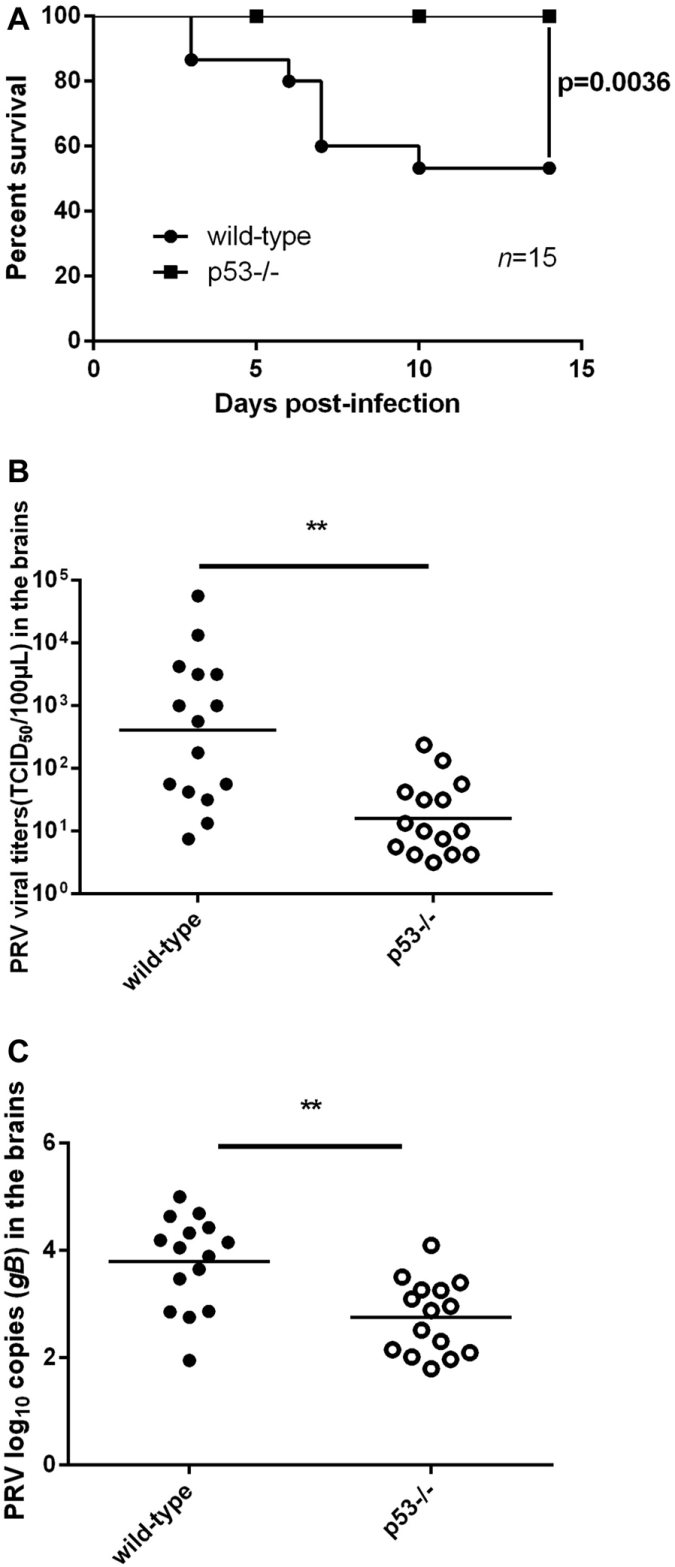
**Effect of p53 on PRV replication in mice brains in vivo**. Four- to 8-week-old male p53^−/−^ mice (*n* = 15) and wild-type mice (*n* = 15) were inoculated with 10^5^ TCID_50_ of PRV (Bartha) intracranially. **A** The survival of the infected p53^−/−^ and wild-type mice was monitored for 14 days post-infection. Statistical significance was determined by the log-rank test. **B**, **C** At 5 days post-infection, the brains of the infected mice were harvested. Viral gB gene copies and virus titers in the brains of the p53^−/−^ and wild-type mice were assayed by quantitative real-time PCR and TCID_50_, respectively. Each data point is the viral titer or viral gB gene copies in the brain of one mouse. The horizontal bars indicate the mean for each group. *P < 0.05; **P < 0.01.

Because PRV infection mainly causes neurological symptoms, in order to investigate the role of p53 in PRV replication in vivo, we detected the mortality, viral load, and viral yield in the brains of wild-type and p53^−/−^ mice. The survival of the infected mice was monitored for 14 days post-infection. As shown in Figure 2A, the survival rate of the p53^−/−^ mice was 100%, while the survival rate of wild-type mice was only 53%, suggesting that p53^−/−^ mice are susceptible to PRV infection. Another group of wild-type and p53^−/−^ mice were infected as described above. The virus titers and viral gB gene in the brains were assayed at 6 days post-infection. The viral titers in the brains of the wild-type mice were significantly higher than those in the brains of the p53^−/−^ mice (Figure 2B). The PRV gB gene copy numbers of the wild-type mice were nearly tenfold that of the p53^−/−^ mice, which was consistent with the viral titers (Figure 2C). The above results indicate that knockout of p53 inhibited PRV replication in mouse brains; this implies that p53 promoted PRV replication in vivo. In summary, these findings further reveal that p53 facilitates PRV viral replication both in vitro and in vivo.

### p53 promoted PRV infection pathogenicity in mice

**Figure 3:**
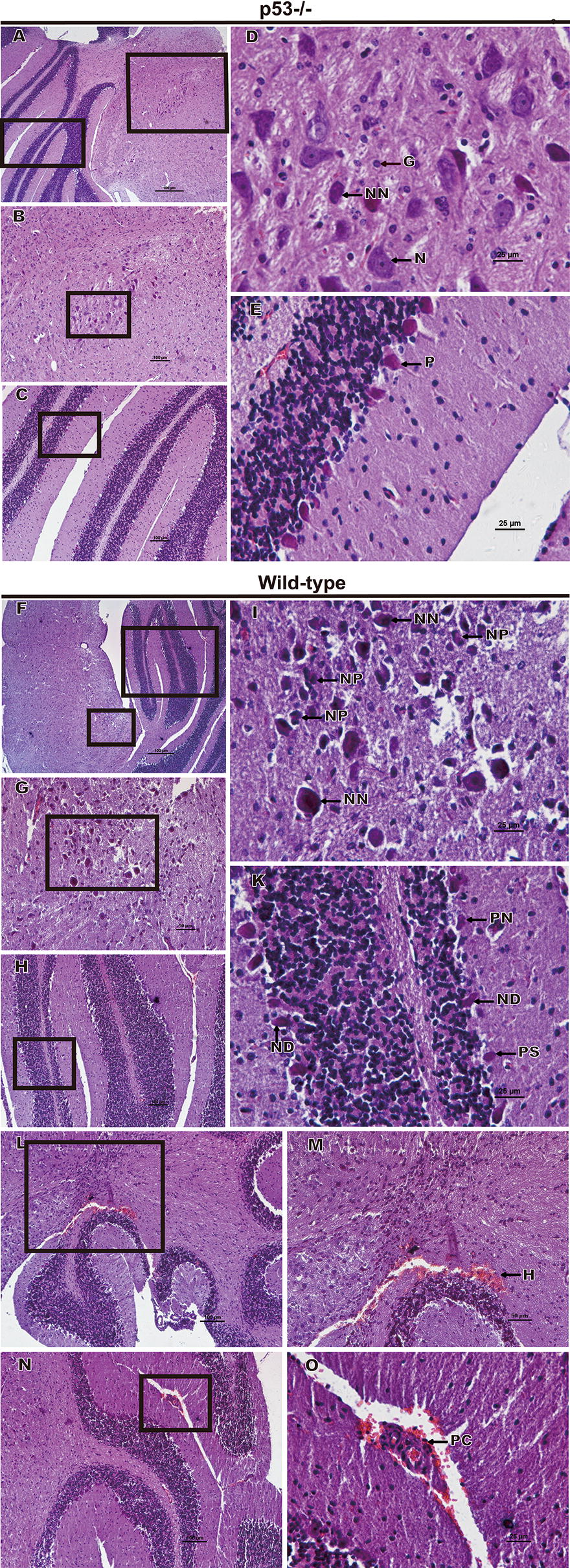
**Histopathological features of the brains of the p53**^**−/−**^ **and wild-type mice following intracranial inoculation.** Four- to 8-week-old male p53^−/−^ (*n* = 7) and wild-type (*n* = 7) mice were inoculated with 10^5^ TCID_50_ of PRV (Bartha) intracranially. At 6 days post-infection, the brains of the infected mice were harvested, sectioned, and stained with hematoxylin and eosin. **B**, **C** Magnified images of the regions indicated with black rectangles in **A**. **G**, **H** Magnified images of the regions indicated with black rectangles in **F**. **D**, **E**, **I**, **K**, **M**, and **O** Magnified images of the regions indicated with black rectangles in **B**, **C**, **G**, **H**, **L**, and **N**, respectively. Representative images are shown. N: neurons; G; glial cells; NN: necrotic neurons; NP: neuronophagia; P: Purkinje cells; PS: shrinkage of Purkinje cells; PN: necrotic Purkinje cells; ND: nuclear disintegration of Purkinje cells; H: hyperemia; and PC: perivascular cuffing.

Encephalitis caused by PRV infection in the CNS is a key factor contributing to animal death. To detect the degree of encephalitis in wild-type and p53^−/−^ mice, we histopathologically analyzed the brains at 6 days post-infection. As shown in Figure 3, the brains of the wild-type mice show a significant increase in the number of necrotic neurons (Figure 3I), and more glial cells were accumulated around the degenerate neurons (glial nodules) than those in p53^−/−^ mice (Figure 3D). Additionally, neuronophagia was found in the brains of the wild-type mice (Figure 3I). In the cerebellum of the wild-type mice, a large number of Purkinje cells show shrinkage or necrosis (Figure 3K) and more obvious nuclear disintegration (Figure 3K) than that in the cerebellum of p53^−/−^ mice (Figure 3E). Furthermore, perivascular cuffing (Figure 3O) and hyperemia (Figure 3M) were more obvious in the brains of wild-type mice.

**Figure 4:**
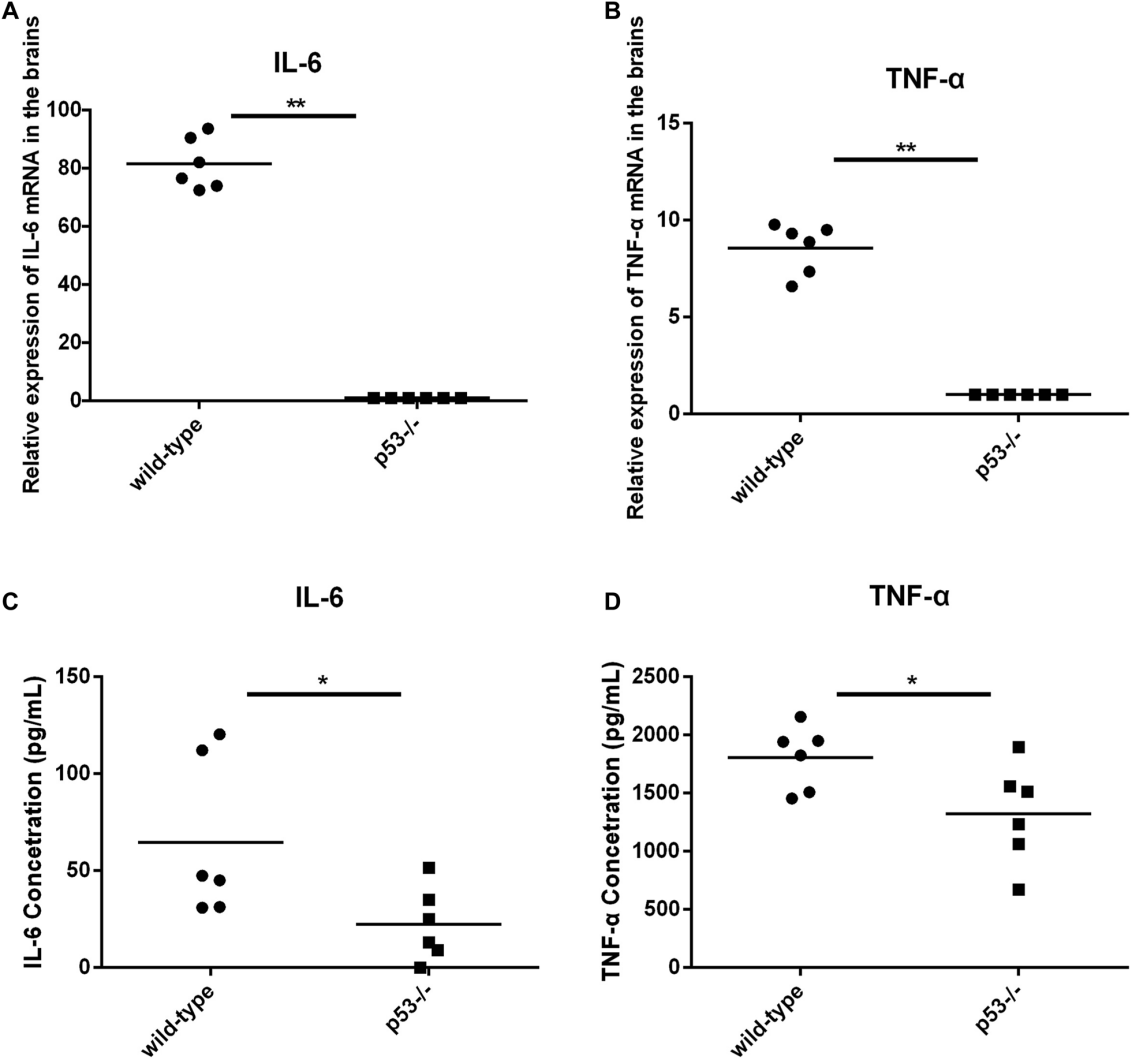
**IL-6 and TNF-α mRNA and antibody levels were detected in the brains of the p53**^**−/−**^ **and wild-type mice following intracranial inoculation.** Four- to 8-week-old male p53^−/−^ (*n* = 6) and wild-type (*n* = 6) mice were inoculated with 10^5^ TCID_50_ of PRV (Bartha) intracranially. At 6 days post-infection, the brains of the infected mice were harvested (**A**–**D**). IL-6 and TNF-α mRNA and antibody levels were detected by relative quantitative real-time PCR and ELISA. Each data point is the level of IL-6 and TNF-α mRNA or antibodies in the brain of one mouse. The horizontal bars indicate the mean for each group. *P < 0.05; **P < 0.01.

We further determined IL-6 and TNF-α mRNA and protein levels in the brains of the wild-type and p53^−/−^ mice by relative quantitative real-time PCR and ELISA. The results show that both IL-6 and TNF-α mRNA expression levels in the brains of the wild-type mice were significantly higher (80-fold and eightfold, respectively) than those in the p53^−/−^ mice (Figures 4A and B). A similar result was also found in protein levels. The protein levels of IL-6 and TNF-α were significantly higher in the brains of the wild-type mice than those in the p53^−/−^ mice (Figures 4C and D); this finding suggests that the wild-type mice experienced a more severe inflammatory response than the p53^−/−^ mice. These results indicate that p53 promotes the pathogenicity of PRV infection in vivo.

### p53 knockout suppresses the inflammatory response in the brains of infected mice

**Figure 5:**
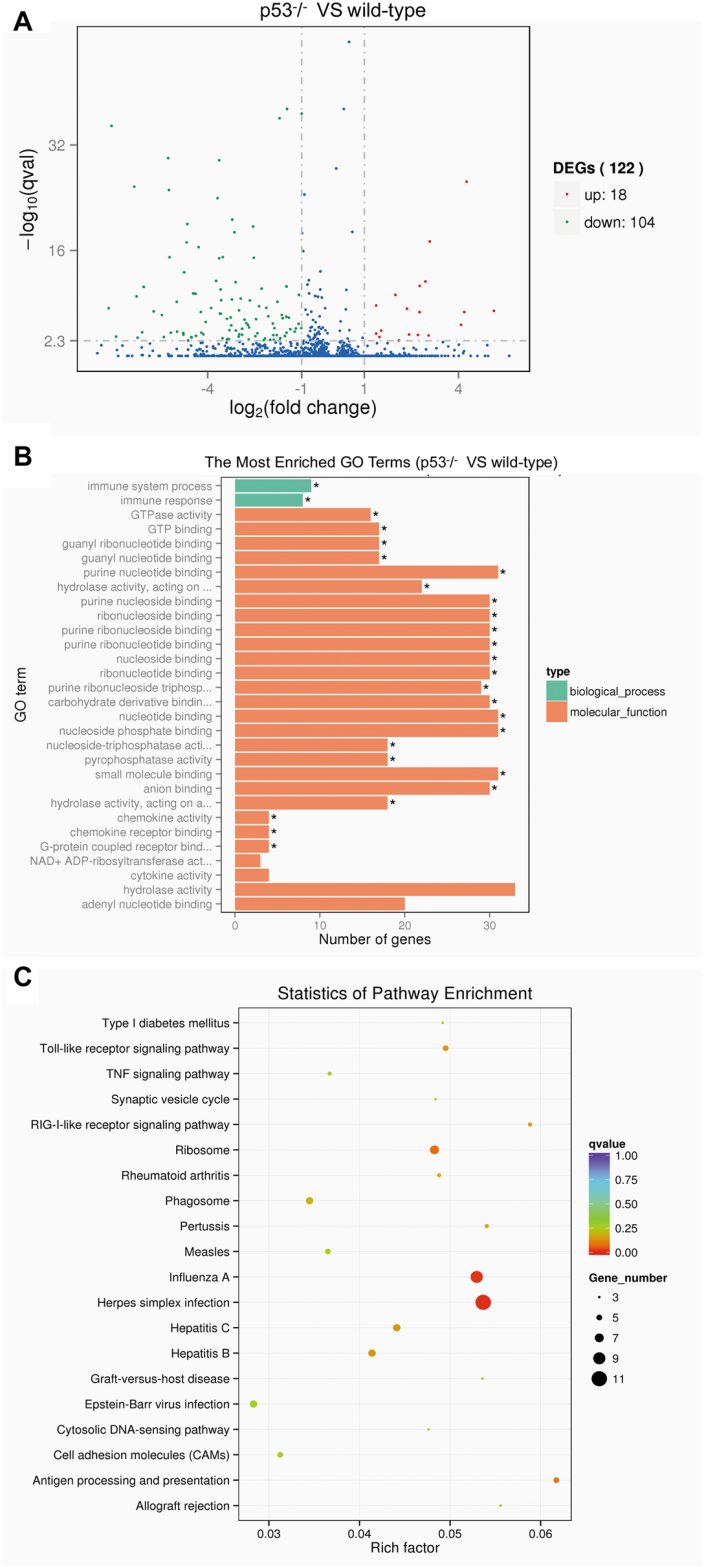
**GO terms and KEGG pathway enrichment analysis of the DEG mRNA. A** The volcano plot for the DEG between the brains of p53^−/−^ and wild-type mice; the x-axis indicates the log fold change and the y-axis indicate the -log (*P* value). **B** The most enriched terms for the differentially expressed mRNA are displayed here. The enrichment score of the GO term equals the -log10 (*P*-value). The listed GO items are categorized as biological processes or molecular functions. **C** The rich factor plot of the KEGG pathway enrichment analysis results. The degree of the color stands for the *P*-value; the size of the node stands for the gene count in this item.

To investigate the effects of p53 knockout on the host cell gene expression in the brains of the infected mice, we performed a whole-transcriptome shotgun sequencing analysis of the brains from the infected p53^−/−^ and wild-type mice following intracranial infection. In the expression profiles, 122 mRNA were differentially expressed, comprising 104 downregulated and 18 upregulated mRNA (Figure 5A). A functional annotation analysis of the target genes of the differentially expressed mRNA was performed to identify the gene ontology (GO) terms and the Kyoto Encyclopedia of Genes and Genomes (KEGG) pathways. As shown in Figure 5B, most of the enriched GO terms of the differentially expressed mRNA were involved in GTPase activity, GTP binding, and guanyl ribonucleotide binding molecular functions as well as the immune response and immune system process. KEGG pathway enrichment analysis indicates that the differentially expressed genes (DEG) tended to be involved in herpes simplex infection, ribosome and antigen processing and presentation as well as TNF signaling and Toll-like receptor signaling pathways (Figure 5C).

Furthermore, the downregulated genes of the DEG were further grouped according to their functions. The results show that compared to the wild-type mice, the major downregulated genes included the IFN-related genes, chemokines, and antiviral genes. As shown in Table 2, the levels of IFN signal pathway-related factors 1 and 7 (IRF1 and IRF7) in the brains of the p53^−/−^ mice were reduced 8.0- and 25.4-fold, respectively, compared to those in the brains of wild-type mice. Further, compared with those in the wild-type mice, the IFN-induced protein family genes (Ifit1, Ifit2, Ifit3, Ifit3b, Ifitm3, Ifip1, Ifih1, Ifi44, and Ifi2712a) in the p53^−/−^ mice were significantly downregulated, with a fold change ranging from 5.7 to 38.2. Similarly, the guanylate-binding protein family genes, which belong to another IFN-induced family, including Gbp 2, Gbp 3, Gbp 4, Gbp 5, Gbp 6, Gbp 7, Gbp 8, Gbp 9, and Gbp 10, were decreased, with a fold change ranging from 6.4 to 38.2. Chemokines are considered to be pro-inflammatory agents and can be induced during an immune response to recruit cells of the immune system to the site of infection. As shown in Table 3, the chemokine gene family, including Ccl2, Ccl7, Cxcl9, and Cxcl10, was found to be downregulated, with a fold change ranging from 44.2 to 133.9. As shown in Table 4, the antiviral genes of the p53^−/−^ mice related to the IFN pathways were downregulated, with fold changes ranging from 3.0- to 76.9-fold; for example, the expression of genes encoding virus entry inhibitors such as Mx1 reduced 76.9-fold and that of genes encoding virus translation and replication inhibitors such as Oasl1 and Oasl2 was reduced 68.0- and 9.3-fold, respectively.

**Table 2:** **Differential expressed of IFN-related genes in brains of PRV-infected p53** ^**−/−**^ **and wild-type mice.**

Gene ID	Gene name	Gene description	Foldchange^a^ (p53^−/−^ vs wild-type)
16362	Irf1	Interferon regulatory factor 1	−8.0
54123	Irf7	Interferon regulatory factor 7	−25.4
15957	Ifit1	Interferon-induced protein with tetratricopeptide repeats 1	−19.6
15958	Ifit2	Interferon-induced protein with tetratricopeptide repeats 2	−12.4
15959	Ifit3	Interferon-induced protein with tetratricopeptide repeats 3	−12.9
667370	Ifit3b	Interferon-induced protein with tetratricopeptide repeats 3b	−10.4
66141	Ifitm3	Interferon induced transmembrane protein 3	−5.7
60440	Iigp1	Interferon inducible GTPase 1	−37.7
71586	Ifih1	Interferon induced with helicase C domain 1	−7.5
99899	Ifi44	Interferon-induced protein 44	−17.6
76933	Ifi27l2a	Interferon alpha-inducible protein 27 like 2A	−9.5
14469	Gbp2	Guanylate binding protein 2	−38.2
55932	Gbp3	Guanylate binding protein 3	−13.3
17472	Gbp4	Guanylate binding protein 4	−31.7
229898	Gbp5	Guanylate binding protein 5	−26.8
100702	Gbp6	Guanylate binding protein 6	−12.0
229900	Gbp7	Guanylate binding protein 7	−6.4
236573	Gbp9	Guanylate binding protein 9	−8.0
626578	Gbp10	Guanylate binding protein 10	−24.6

^a^ Fold activation represents the fold increase in the level of activation in p53^−/−^ mice compared with the level of activation in wild-type mice.

**Table 3:** **Differential expressed chemokines in brains of PRV-infected p53** ^**−/−**^ **and wild-type mice.**

Gene ID	Gene name	Gene description	Foldchange^a^ (p53^−/−^ and wild-type)
20296	Ccl2	Chemokine (C–C motif) ligand 2	−65.7
20306	Ccl7	Chemokine (C–C motif) ligand 7	−42.2
17329	Cxcl9	Chemokine (C-X-C motif) ligand 9	−75.1
15945	Cxcl10	Chemokine (C-X-C motif) ligand 10	−133.9

^a^ Fold activation represents the fold increase in the level of activation in p53^−/−^ mice compared with the level of activation in wild-type mice.

**Table 4:** **Differential expressed anti-viral genes in brains of PRV-infected p53** ^**−/−**^ **and wild-type mice.**

Gene ID	Gene name	Gene description	Foldchange^a^ (p53^−/−^ and wild-type)
20846	Stat1	Signal transducer and activator of transcription 1	−3.0
53817	Ddx39b	DEAD (Asp-Glu-Ala-Asp) box polypeptide 39B	−14.8
230073	Ddx58	DEAD (Asp-Glu-Ala-Asp) box polypeptide 58	−5.6
20210	Saa3	Serum amyloid A 3	−44.0
110454	Ly6a	Lymphocyte antigen 6 complex locus A	−3.8
100041546	Ly6c2	Lymphocyte antigen 6 complex locus C2	−23.0
231655	Oasl1	2′-5′ oligoadenylate synthetase-like 1	−68.0
23962	Oasl2	2′-5′ oligoadenylate synthetase-like 2	−9.3
17857	Mx1	MX dynamin-like GTPase 1	−76.9
58185	Rsad2	Radical S-adenosyl methionine domain containing 2	−37.1

^a^ Fold activation represents the fold increase in the level of activation in p53^−/−^ mice compared with the level of activation in wild-type mice.

**Figure 6:**
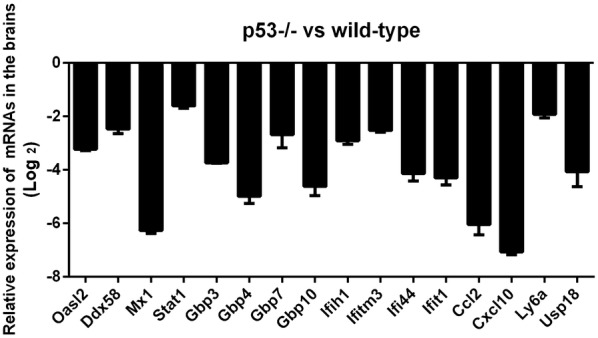
**The expression levels of the 16 downregulated genes of the p53-/- mice were detected by relative quantitative real-time PCR.** Oasl2, Ddx58, Mx1, Stat1, Gbp3, Gbp4, Gbp7, Gbp10, Ifih1, lfitm3, Ifi44, Ifit1, Ccl2, Cxcl10, Ly6a and Usp18 were detected by relative quantitative real-time PCR to validate the data obtained by RNA sequencing analysis. *P < 0.05; **P < 0.01.

To validate the data obtained by RNA sequencing analysis, 16 downregulated genes were selected and their expression was detected by relative quantitative real-time PCR. As shown in Figure 6, the expression levels of the 16 genes (Oasl2, Ddx58, Mx1, Stat1, Gbp3, Gbp4, Gbp7,Gbp10, Ifih1, lfitm3, Ifi44, Ifit1, Ccl2, Cxcl10, Ly6a, Usp18) in the p53^−/−^ mice were significantly decreased compared to those in the wild-type mice. These results were consistent with the findings of RNA sequencing analysis.

## Discussion

PRV causes encephalitis that can result in severe neurological defects and death in swine. Many of the host cell factors involved in the regulation of PRV infection have been investigated. However, most of these factors are immunological regulators and function through immunological pathways to restrict PRV infection. These factors therefore provide limited information on the intrinsic host cell regulators that may be involved in the facilitation of PRV infection. Here, we demonstrate that a host cell protein, p53, which has generally been considered as a host cell restriction factor for various viral infections, is required for efficient PRV replication and pathogenesis in mice. This is the first report showing that p53 positively regulates PRV replication and pathogenesis in vitro and in vivo and provides insights into the molecular mechanism of p53.

Animals usually have many defense strategies against viral infection; however, viruses have evolved complex tactics to override these lines of defense, for instance, by using the host defensive proteins to promote viral replication [14–16]. p53 is a critical host restriction factor because its regulation of the various cellular life processes, such as cell cycle arrest, apoptosis, and autophagy [6], has both positive and negative effects on various viral infections [10, 12]. On the basis of previous studies, double-stranded RNA are produced in RNA virus infections, and these double-stranded RNA trigger antiviral responses mediated by type I interferon (IFN-I) signaling, in which p53 appears to reduce the replication of some viruses; therefore, these viruses have acquired mechanisms to counteract p53 in infected cells [7, 8]. However, in DNA virus infection, viral genome replication induces host DNA damage responses (DDRs), which activate apoptotic p53 responses [6, 10]. In agreement with these observations, we found that p53 overexpression promoted PRV replication, whereas inhibiting p53 by a specific inhibitor reduced PRV replication in PK-15 cells in vitro. p53^−/−^ mice were also not susceptible to PRV infection compared to wild-type mice in vivo. Following intracranial inoculation, p53 knockout reduced viral replication in the brains of mice and led to significantly reduced rates of mortality. However, PRV titers and viral gene copy numbers in the brain as well as mortality after intracranial inoculation appear to be surprisingly low. The possible reason is that the attenuated PRV vaccine strain (PRV-Bartha) which was used for intracranial inoculation in our studies. Brittle et al. [17] reported that mice infected with an attenuated PRV vaccine strain (PRV-Bartha) survive approximately three times longer than virulent (e.g., PRV-Becker, PRVKaplan, or PRV-NIA3) strain-infected mice. Furthermore, they indicated that the absence of the genes encoding US9, gE and gI in PRV-Bartha accounts for much of its attenuation. The absence of US9, gE and gI genes led to the time delay of PRV-Bartha entering various tissue types. In addition, the amount of infectious PRV-Bartha in the brainstem was lower relative to virulent PRV at the equivalent time point. Taken together, these results suggest that p53 has a positive effect on PRV replication both in vitro and in vivo.

It has previously been reported that PRV invades and spreads within the trigeminal pathway (the nasal mucosa, the trigeminal ganglion, the pons/medulla, and the cerebellum/thalamus) of neonatal pigs [18, 19]. PRV induces encephalitis in both pigs and mice with similar pathological signs [18, 20]. Although it was reported that the attenuated Bartha strain does not cause severe brain pathology despite viral replication and spread throughout the brain in chicken embryos [21], our histopathology results show much more serious encephalitis in the brains of wild-type mice than in those of the p53^−/−^ mice. Compared to the p53^−/−^ mice, wild-type mice show neuronal degeneration and necrosis in the brain. Notably, Purkinje cells in the cerebellum of wild-type mice show more obvious shrinkage or necrosis and nuclear disintegration than those in p53^−/−^ mice. These histolpathological changes provided morphological data to support our observation of serious neurological signs, including convulsion, ataxia, and abnormal behavior, in wild-type mice after PRV infection. Our findings coincided with the conclusion of previous studies that PRV infection impairs cerebellar development and differentiation [18, 20]. Furthermore, two cytokines associated with inflammation, namely IL-6 and TNF-α, in the brains of the p53^−/−^ mice were also significantly lower than those in the brains of wild-type mice; this finding coincided with the development of encephalitis. These results indicate that p53 is required for efficient virulence and replication of the virus and for the consequent development of viral encephalitis in the brains of mice following intracranial inoculation. This requirement of p53 for efficient viral replication in the brains of mice is in agreement with the findings of our in vitro study using cell culture, as described above. To our knowledge, this is the first report showing that p53 plays a positive role in PRV replication and pathogenesis both in vitro and in vivo.

We further investigated mRNA profiles in the PRV-infected p53^−/−^ and wild-type mice by RNA sequencing. The gene function analysis shows that differentially expressed RNA enrichment was observed in a number of the pathways in the host antiviral immune response and the inflammatory response processes. The DEG primarily enriched the interferon-related pathways, including interferon-regulated genes (IRG), interferon-inducible protein family genes, interferon-inducible guanylate-binding protein family genes (Gbp), and interferon-stimulated genes (ISG). Interferon is an important antiviral factor of the body [22, 23]. It has been reported that alpha/beta interferon receptor deficiency in mice significantly enhanced their susceptibility to PRV infection [24]. In molecular biology, interferon-inducible protein family genes, namely Gbps and ISG, are key factors for protective immunity against microbial and viral pathogens [25–27]. The expression of all the above mentioned genes was significantly downregulated in the p53^−/−^ mice compared with that in wild-type mice, suggesting that the PRV replication progress in the p53^−/−^ mice was inhibited. Moreover, some of the cytokine ligands and anti-viral genes show significantly different expression between the p53^−/−^ and wild-type mice. Notably, Cxcl10, Oasl1, and Ccl7 in the brain of p53^−/−^ mice were reduced by 133-, 67-, and 42-folds, respectively, compared with those in wild-type mice. Previous studies have demonstrated that Cxcl10 and Ccl7 can offer protective immunity against PRV, while OASL1 deficiency promotes antiviral immunity against local mucosal viral infection [28–30]. These data corroborate our suggestion that p53 promotes PRV viral replication and pathogenesis in mice.

In conclusion, this study provides evidence suggesting that p53 positively regulates viral replication and pathogenesis both in vitro and in vivo. We demonstrate that the host cell protein p53 can be a host cell restriction factor for PRV infection. Our study offers novel therapeutic targets of intrinsic host cell immunity for PRV infection.
